# Emulsion-Based Encapsulation of Fibrinogen with Calcium Carbonate for Hemorrhage Control

**DOI:** 10.3390/jfb16030086

**Published:** 2025-03-03

**Authors:** Henry T. Peng, Tristan Bonnici, Yanyu Chen, Christian Kastrup, Andrew Beckett

**Affiliations:** 1 Defence Research and Development Canada, Toronto Research Centre, Toronto, ON M3K 2C9, Canada; tristan.bonnici@drdc-rddc.gc.ca; 2 Department of Nanotechnology Engineering, University of Waterloo, Waterloo, ON N2L 3G1, Canada; 3 Michael Smith Laboratories and Department of Biochemistry and Molecular Biology, University of British Columbia, Vancouver, BC V6T 1Z4, Canada; 4 Versiti Blood Research Institute, Milwaukee, WI 53226, USA; ckastrup@versiti.org; 5 St. Michael’s Hospital, University of Toronto, Toronto, ON M5B 1W8, Canada; andrew.beckett@unityhealth.to; 6 Royal Canadian Medical Services, Ottawa, ON K1A 0K2, Canada

**Keywords:** calcium carbonate, hemorrhage control, hemostatic particle, fibrinogen, self-propulsion, tranexamic acid, trauma

## Abstract

Hemorrhage, particularly non-compressible torso bleeding, remains the leading cause of preventable death in trauma. Self-propelling hemostats composed of thrombin-calcium carbonate (CaCO_3_) particles and protonated tranexamic acid (TXA^+^) have been shown to reduce blood loss and mortality in severe bleeding animal models. To further enhance both hemostatic and self-propelling properties, this study was to investigate fibrinogen-CaCO_3_ particles prepared via a water-oil-water (W/O/W) emulsion method. The particles were characterized using light and fluorescence microscopy, gel electrophoresis, rotational thromboelastometry (ROTEM), and video motion tracking. The method produced spherical micrometer-sized particles with various yields and fibrinogen content, depending on the preparation conditions. The highest yield was achieved with sodium carbonate (SC), followed by ammonium carbonate (AC) and sodium bicarbonate (SBC). AC and paraffin generated smaller particles compared to SC and heptane, which were used as the carbonate source and oil phase, respectively. Fibrinogen incorporation led to an increase in particle size, indicating a correlation between fibrinogen content and particle size. Fluorescence microscopy confirmed successful fibrinogen encapsulation, with various amounts and hemostatic effects as assessed by gel electrophoresis and ROTEM. Combining fibrinogen-CaCO_3_ particles with TXA^+^ and thrombin-CaCO_3_ particles showed synergistic hemostatic effects. All fibrinogen-encapsulated particles exhibited self-propulsion when mixed with TXA^+^ and exposed to water, regardless of fibrinogen content. This study advances current hemostatic particle technology by demonstrating enhanced self-propulsion and fibrinogen incorporation via the W/O/W emulsion method. Further optimization of the encapsulation method could enhance the effectiveness of fibrinogen-CaCO_3_ particles for hemorrhage control.

## 1. Introduction

Bleeding from injuries, surgical procedures, and disease- or drug-associated blood disorders can result in significant mortality and morbidity [1]. Despite significant advances in hemostatic materials and blood products, uncontrolled bleeding remains the leading cause of preventable death in combat trauma, accounting for 90% of fatalities [2,3]. It is also the primary cause of preventable death in 30–40% of the six million trauma victims each year, with half dying in the pre-hospital setting [4,5,6]. Notably, non-compressible torso hemorrhage contributes to over 60% of these preventable deaths [7]. Effective hemorrhage control during pre-hospital and early in-hospital time frames is critical for reducing trauma mortality. Furthermore, major hemorrhage is a significant cause of morbidity following cardiac surgery and liver transplantation, and it ranks among the most common causes of death in women during delivery [8].

Hemostatic materials have been investigated for prehospital control of life-threatening hemorrhage [9,10,11]. They have been developed for application at the bleeding site, including silicate-based inorganic materials (such as zeolite, kaolin, and smectite) and polymer-based organic materials (like chitosan) [12,13,14]. These hemostatic agents come in various forms, including fabric, sponge, gel, powder, foam, and flowable liquids, utilizing both biologically derived and synthetic materials [12,15,16]. Key components include proteins (fibrin, fibrinogen thrombin, gelatin, collagen) [17,18], polysaccharides (cellulose, alginate, starch, and chitosan) [18,19], peptides [18] and synthetic composites [20]. These hemostats facilitate hemorrhage control through biochemical and mechanical mechanisms, such as triggering thrombin generation, accelerating platelet activation, promoting blood absorption and fibrin accumulation, aggregating coagulation factors and blood cells, and reducing fibrinolysis. The integration of these mechanisms is essential for effective hemostatic action, particularly in critical situations where rapid and reliable hemorrhage control can significantly impact patient outcomes.

The development of effective hemostatic agents is crucial in emergency settings, especially for acute hemorrhage where rapid intervention can save lives [11]. Current hemostatic dressings, such as Combat Gauze and HemCon, rely on manual pressure to achieve hemostasis, which is not ideal in high-stress environments like combat or for internal bleeding [21]. These agents often struggle to reach deep tissue injuries, and their non-biodegradable nature necessitates removal post-application, risking re-bleeding [22].

Recent advancements in hemostatic agents, particularly the use of nano- and micro-sized powders, show promise in overcoming these limitations [23]. Due to their nano/micro sizes, hemostatic agents in powder form are not restricted by the size, shape, or location of a wound, making them suitable for irregular, deep, or internal injuries [24]. Their large surface area-to-volume ratio and porous structure enable strong fluid absorption, water swelling, and extensive contact with hemorrhagic sites. This enhances activation of the clotting cascade, blood cell adhesion, and overall biodegradability and safety in medical applications [25,26]. Furthermore, biodegradable hemostatic powders can be endoscopically delivered to stop gastrointestinal bleeding [27] or control other significant hemorrhages during minimally invasive surgeries, such as laparoscopy, helping to reduce surgical complications. Hemostatic microspheres containing biological macromolecules further improve these properties [23].

However, a major limitation of traditional hemostatic powders is their tendency to be displaced by the pressurized blood flow from the wound, preventing proper delivery and delaying clot formation at the site of injury. This challenge is particularly evident when bleeding results from severe trauma, when damaged vessels cannot be located, or when compression is not possible [28]. Unlike bulk-form hemostats (e.g., sponges or membranes), powder particles lack active driving forces or targeted guidance, making them prone to drifting passively with dynamic blood flow [29]. As a result, the underlying bleeding site remains insufficiently treated, and the hemostatic agents may only function on the wound’s surface. This issue has persisted as a significant challenge in hemostasis.

To address this, self-propelling hemostatic particles have been developed [28]. These particles, composed of thrombin-bound CaCO_3_ and protonated tranexamic acid (TXA^+^), utilize a neutralization reaction to generate CO_2_ gas upon contact with blood, propelling the hemostatic agents directly into the injury site. TXA is an approved antifibrinolytic drug that prevents the resultant clot from fibrinolysis when administered early systematically or applied locally [30]. Therefore, it makes the self-propelling particle more effective at stopping hemorrhage. As a by-product of this neutralization reaction, the calcium ion may further promote coagulation and prevent hypocalcemia [31]. All these features work synergistically for the most effective hemostasis.

Additionally, incorporating fibrinogen into these particles is crucial, given its role as an essential clotting factor and association of low fibrinogen levels with increased bleeding, coagulopathy and poor clinical outcomes [32]. Traditional formulations may not fully deliver coagulation factors to the site of bleeding due to weak interactions. Encapsulating fibrinogen within the CaCO_3_ particles could improve delivery efficiency and hemostatic effectiveness.

In this paper, we present the preparation and characterization of a novel hemostatic material composed of fibrinogen-encapsulated CaCO_3_ particles. These particles were created using a water-oil-water (W/O/W) emulsion method under various conditions. We evaluated their properties through light and fluorescence microscopy to assess particle size and fibrinogen distribution, gel electrophoresis to quantify the encapsulated fibrinogen, rotational thromboelastometry (ROTEM) to investigate hemostatic effects, and video motion tracking for examine self-propelling capabilities. This formulation of fibrinogen-encapsulated CaCO_3_ particles has the potential to enhance hemostatic materials for managing severe bleeding.

## 2. Materials and Methods

### 2.1. Materials

Ammonium carbonate (AC, ACS grade), sodium carbonate (SC, 98% purity), sodium bicarbonate (SBC, >99% purity), calcium chloride (CaCl_2_, anhydrous, ≥96% purity), 4-(2-hydroxyethyl)-1-piperazineethanesulfonic acid (HEPES, 99% purity), paraffin (pure liquid), heptane (ACS grade), Span 80, Tween 80 (for Biochemical Research), tranexamic acid (>98% purity), polyethylene glycol (PEG, molecular weight of 6000), polyvinyl alcohol (PVA, 98–99% hydrolyzed, low-molecular-weight), fluorescein isothiocyanate (FITC), Invitrogen™ Novex™ tris-glycine mini protein gels (8–16%, 1.0 mm, WedgeWell™ format), iBright™ prestained protein ladder, sodium dodecyl sulfate (SDS) buffer, dithiothreitol (DTT, 99.5% purity), coomassie blue (SimplyBlue™ SafeStain, Fisher Scientific (Ottawa, ON, Canada)), and Invitrogen™ Triton X-100 (1%) were purchased from Fisher Scientific (Ottawa, ON, Canada). Fibrinogen concentrate was obtained from CSL Behring (King of Prussia, PA, USA). Citrated human plasma with an abnormally low concentration of fibrinogen was purchased from Precision BioLogic Inc. (Dartmouth, NS, Canada).

### 2.2. Preparation of Self-Propelling Particles

The particles are mixed fibrinogen-CaCO_3_ particles and TXA^+^. The former was prepared by the emulsion-based encapsulation method detailed below.

#### 2.2.1. Water-Oil-Water Emulsion Method

The fibrinogen-CaCO_3_ particle was prepared using an interfacial reaction method with a W/O/W emulsion, as reported [33]. Typically, in one beaker, 1.153 g of AC was dissolved in 4 mL of Milli-Q water, while in another, 14.3 mg of HEPES was dissolved in 3 mL of Milli-Q water. Subsequently, 105 mg fibrinogen was added for reconstitution. Once fully reconstituted, the carbonate solution was slowly added to the fibrinogen-HEPES solution under constant stirring. A mixture of 14 mL heptane, 41.3 mg Span 80 and 83.7 mg Tween 80 was homogenized in a glass vial using a high-speed vortex for 1 min. This heptane-surfactant emulsion was combined with the fibrinogen-carbonate solution to create a water/oil mixture. The resulting mixture was stirred using a magnetic stirring bar at 990 rotations per minute (RPM) for 5 min. Finally, this emulsion mixture was added to 80 mL of 0.3 M CaCl_2_ aqueous solution and stirred at 400 RPM for 10 min to produce fibrinogen-encapsulated CaCO_3_ particles. In some preparations, polymers (PEG and PVA) were included in the fibrinogen-carbonate solution to protect fibrinogen from denaturation and stabilize the W/O/W emulsion. These polymers, combined with surfactants, help form surfactant-polymer templates for the precipitation of CaCO_3_ particles [34]. Control particles, which did not contain fibrinogen, were also prepared under identical conditions. The resulting precipitated particles were collected by centrifugation at 3000 RPM for 10 min and washed three times with Milli-Q water before being lyophilized under vacuum. Thrombin-CaCO_3_ particles (Enc Thr) were also prepared by the encapsulation method as previously reported [33].

As shown in Table 1, various conditions were investigated, including different carbonate types (AC, SC, SBC) and AC concentrations (1, 2 and 3 M), fibrinogen concentrations (15 and 20 mg/mL), oil phases (heptane and paraffin), reaction time (10 and 30 min), amounts of Span 80 and Tween 80, additions of PEG and PVA, and switch of the internal and external water phase between AC and CaCl_2_.

#### 2.2.2. Protonation of TXA

Protonated TXA (TXA^+^) was prepared by first dissolving TXA at its unprotonated form into Milli-Q water at 10% weight-to-volume ratio. One milliliter of stock hydrochloric acid (HCl) was then added into the solution to achieve a pH of approximately 4.5. Any necessary fine tuning of the pH was conducted by adding 100 µL of HCl at a time to lower the pH, or sufficient water to raise it. The target pH of the solution was set at 4.3, as described elsewhere [35]. Finally, the solution was lyophilized under vacuum to obtain solid TXA in its protonated form, TXA^+^.

### 2.3. Characterization of Fibrinogen-Encapsulated CaCO_3_ Particles

The morphologies of fibrinogen-encapsulated CaCO_3_ particles were characterized using light and fluorescence microscopy. To assess their suitability for hemorrhage control, we measured the hemostatic and self-propelling properties of the particles through various methods outlined below. The presence and hemostatic effects of fibrinogen were quantified by gel electrophoresis and ROTEM, both of which have been established for analyzing fibrinogen content [36]. The self-propelling ability of the particles mixed with TXA^+^ was evaluated using real-time video recording, which allowed for quantitative analysis of their response time and movement speed, following previously reported methods [37].

#### 2.3.1. Light and Fluorescence Microscopy

Microscopy images were acquired by ZEISS LSM 800—Airyscan monitored by ZEN BLUE (Carl Zeiss Canada Ltd., North York, ON, Canada) for all synthesized particles. A small sample of particles was spread onto a glass slide and gently shaken to remove any excess material.

##### FITC Labeling

FITC-labeled proteins were prepared to validate encapsulation within CaCO_3_ particles. To prepare FITC-labeled fibrinogen, a FITC stock solution was prepared by dissolving 10 mg of FITC in 1 mL of dimethylformamide and diluting it tenfold. An optimal concentration of FITC solution to fibrinogen was then added to the prepared fibrinogen-carbonate solution at a 0.15 molar ratio. The molar ratio was determined through testing, as higher concentrations of FITC caused fibrinogen to denature and form a gel-like structure during the labeling reaction. The resulting mixture was incubated in darkness for 30 min while being stirred at 200 RPM. The labeled fibrinogen solution was then used in the preparation of FITC-labeled fibrinogen-CaCO_3_ particles through the aforementioned method. FITC-labeled samples underwent two additional washes with isopropanol during centrifugation to eliminate any excess FITC dyes adhering to the outer layer of CaCO_3_ particles. A control of FITC-labeled particles without fibrinogen was prepared to guide further corrections for excess FITC residue.

##### Imaging Analysis

The initiation of the fluorescent effect of the particles was facilitated by the 488 nm laser module, and all wavelengths from 488 to 620 nm were captured by detectors. The specific imaging parameter, determined through trial and error to produce the clearest imaging, was consistently maintained for all instances, allowing comparison between similar samples if the parameter remains constant.

#### 2.3.2. Gel Electrophoresis

Particle samples weighing 10 mg were rocked in a 0.5 mL pH 4.3 TXA^+^ solution for 2 h to dissolve CaCO_3_ and release fibrinogen. The samples were then centrifuged, and the obtained supernatants (10 µL) were mixed with 30 µL of SDS buffer, 40 µL of Milli-Q water, and 8–10 mg of DTT as a reducing agent. Fibrinogen samples were prepared at concentrations of 0.04, 0.2, 1 and 5 mg/mL in TXA^+^ solution to service as standards and positive control. The mixture was heated at 90 °C for 5 min and analyzed by continuous SDS-polyacrylamide gel electrophoresis (SDS-PAGE) on an Invitrogen™ Mini Gel Tank (Fisher Scientific, Ottawa, ON, Canada). The gel was run at 125 V and 50 mA for 100 min, then stained with coomassie blue and washed in Milli-Q water overnight. A prestained protein ladder, composed of a mixture of 12 proteins ranging from 11 to 250 kDa, was included as molecular weight standards (Mw STD).

The relative optical intensity of each band was quantified by densitometric analysis using the program ImageJ 1.54j downloaded from https://imagej.net/ij/index.html [38] (accessed on 15 June 2024). Specifically, the fibrinogen content in the CaCO_3_ particle samples was estimated using a standard curve showing a linear correlation between the band intensity volume (peak area) and fibrinogen standards of known concentrations in a range of 0.04 to 5 mg/mL.

#### 2.3.3. Rotational Thromboelastometry (ROTEM)

ROTEM has been widely used to measure the hemostatic effects of materials in various forms [39]. In this study, ROTEM was conducted with particles to evaluate their impact on hemostasis using citrated human plasma with an abnormally low fibrinogen concentration, at 37 °C on a ROTEM Delta machine (Instrumentation Laboratory, Bedford, MA, USA). Typically, 6 mg of fibrinogen-containing CaCO_3_ or blank CaCO_3_ particles were added to a ROTEM cup, followed by 20 µL of star-tem (0.2 M CaCl_2_) and 300 µL of plasma, in accordance with the manufacturer’s standard procedure for the NATEM test. Additionally, NATEM tests were performed with a combination of 6 mg of fibrinogen-CaCO_3_ particles, 2 mg of thrombin-CaCO_3_ particles, and 2 mg of TXA^+^, all pre-mixed in a ROTEM cup, followed by the same procedure. All tests were run for at least 60 min to measure coagulation time (CT) and maximum clot firmness (MCF).

#### 2.3.4. Hemolysis Test

Red blood cells (RBCs) were isolated by centrifuging blood samples from healthy volunteers for 10 min at 6000 RPM, followed by extraction of the supernatant from each tube. The RBCs were then washed three times with phosphate-buffered saline (PBS), pH 7.4, and suspended in PBS at a final concentration of 5% (volume/volume). The blood collection protocol was approved on 25 April 2023 by the Human Research Ethics Committee of Defence Research and Development Canada (#2022-037). All volunteers provided informed consent.

To assess blood compatibility, each particle sample was dispersed in PBS at a concentration of 2 mg/mL and mixed with an equal volume of the RBC suspension. The mixture was incubated at 37 °C for 1 h, then centrifuged for 5 min at 2500 RPM. The supernatant was extracted and diluted 10-fold with PBS. Absorbance was measured at 540 nm using a UV-Visible spectrophotometer (Agilent Technologies Canada Inc., Mississauga, ON, Canada). PBS and 1% Triton X-100 were used as negative and positive controls.

The percentage of hemolysis was calculated using the following equation: hemolysis (%) [(A_s_ − A_n_)/(A_p_ − A_n_)] × 100%, where A_s_, A_n_, and A_p_ are the absorbance of the sample, negative control and positive control, respectively.

#### 2.3.5. Self-Propulsion Test

Fibrinogen-loaded or unloaded CaCO_3_ particles and TXA^+^ were mixed in a 12:10 mass ratio (12 mg Fibrinogen-CaCO_3_ particles and 10 mg TXA^+^). The mixture was aliquoted into approximately 2 mg piles on an aluminum weigh boat. A 1 mL pipette was filled with 0.6 mL of water, sealed with parafilm, and the open end was applied to the sample. The particles were typically observed self-propelling up the length of the pipette.

To quantify the movement of these particles over time, Tracker, a motion-tracking program available at https://opensourcephysics.github.io/tracker-website/ (accessed on 15 June 2024), was used. Two primary metrics were derived from the self-propulsion tracking: lag time, measured in seconds, indicates the duration from when the pipette tip encounters the sample to the movement of the tracked particle. The second metric, speed, measured in cm/second, represents the speed of the particle over the first 20 data points collected after its initial separation from the weigh boat.

#### 2.3.6. Statistical Analysis

Data points were expressed as mean ± standard deviation (SD) (n = 3) unless otherwise specified. Intergroup analyses were conducted using independent t tests. Pearson correlation analyses were performed to evaluate relationships between fibrinogen content and particle size, as well as their respective relationships with ROTEM and self-propelling measurements. Cohen’s criteria were applied to assess the strength of the correlations: r = 0.1–0.3 (low), r = 0.3–0.5 (moderate), r = 0.5–1.0 (high) [40]. All statistical analyses were conducted using SPSS Statistics 28 (IBM Corporation, Armonk, NY, USA), and a *p*-value of less than 0.05 was considered significant.

## 3. Results

### 3.1. Particle Yield and Fibrinogen Content

As summarized in Table 1, SC resulted in the highest yield above 75%, followed by AC around 40%, SBC at 21% yield when either heptane or paraffin was used in the W/O/W encapsulation method. When AC was used, the yield increased from 36% to 50% as its concentration increased from 1 M to 3 M. Increasing fibrinogen concentration from 0 (NoFib) to 20 mg/mL decreased the yield from 51% to 39%, and 46% to 41% when heptane and paraffin were used as the oil phase, respectively. When heptane was used as the oil phase, it could produce a higher yield than paraffin (e.g., 50% for batch Enc AC Fib Hep versus 43% for batch Enc AC Fib Par). Increasing the reaction time from 10 to 30 min increased the yield from 41% to 45% (Enc AC Fib_high_ Par versus Enc AC Fib_high_ Par 30 min). Doubling the amount of surfactants slightly increased the yield from 40% to 43%. The addition of polymers (PEG and PVA) reduced the yield from 50% to approximately 40%. Switching the inner water phase from AC to CaCl_2_ resulted in the largest increase in the yield from 40% to 70% (Enc AC_low_ Fib Hep versus Enc CaCl_2_ Fib Hep AC).

Table 1 also summarizes the fibrinogen content encapsulated in the CaCO_3_ particles prepared under various conditions. Larger fibrinogen contents were obtained using AC as the carbonate source, a higher fibrinogen concentration (15 mg/mL versus 20 mg/mL), heptane as the oil phase, a larger amount of surfactants (Enc AC_low_ Fib Hep Surf_high_ versus Enc AC_low_ Fib Hep), the addition of PEG (Enc AC Fib Hep PEG versus Enc AC Fib Hep), and by switching the internal water phase from AC to CaCl_2_, respectively, in the preparation. No clear increase in the fibrinogen content was observed with increasing the AC concentration from 1 M to 3 M. It should be noted that the preparation condition may have opposite effects on the particle yield versus fibrinogen content.

### 3.2. Particle Morphology and Size

**Figure 1:**
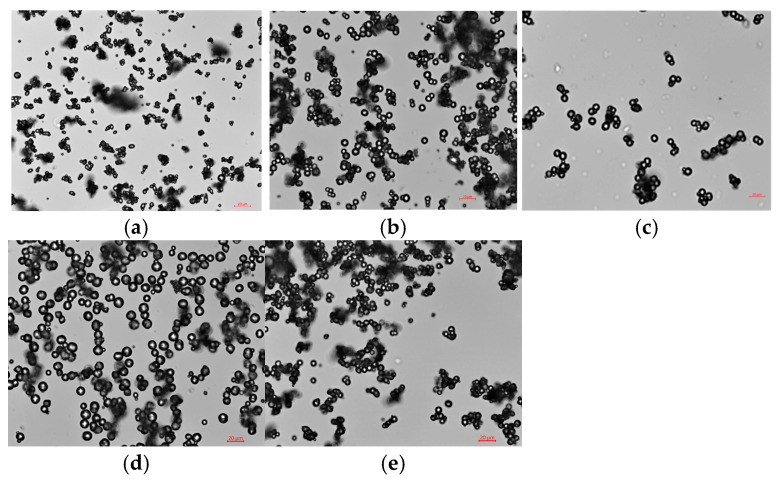
Images of light microscopy of (**a**) Enc AC_lowest_ Fib Hep, (**b**) Enc AC_low_ Fib Hep, and (**c**) Enc AC Fib Hep, (**d**) Enc AC Fib_high_ Hep and (**e**) Enc AC Fib Par. The scale bar represents 20 µm. See Table 1 for details of the sample preparation.

Figure 1 shows the light microscopy images of fibrinogen-encapsulated CaCO_3_ particles prepared with varied concentrations of AC (1, 2 and 3 M), fibrinogen (15 and 20 mg/mL) and oil phase (heptane and paraffin). All particles exhibit nearly spherical structures with bright central regions surrounded by dark edges, and take the form of spheres coalesced into aggregations. The particle size increased with AC concentrations (Figure 1a–c). The particles prepared with a higher fibrinogen concentration and heptane as the oil phase appeared larger, respectively (Figure 1c versus Figure 1d and Figure 1c versus Figure 1e).

As summarized in Table 2, the particles were micrometers in diameter ranging from 2.7 (Enc AC NoFib Hep) to 10.8 µm (Enc AC Fib_high_ Hep). As AC concentration increased from 1 M to 3 M, the particle diameter increased approximately from 4.6 µm to 9.9 µm. At the same concentration (1 M), SC produced larger particles than AC and SBC (7.6 µm versus 4.6 µm and 5.2 µm) perhaps due to faster reaction with CaCl_2_ and crystallization/growth of the resultant CaCO_3_ in agreement with higher yield obtained with SC than AC and SBC (Table 1).

**Figure A1:**
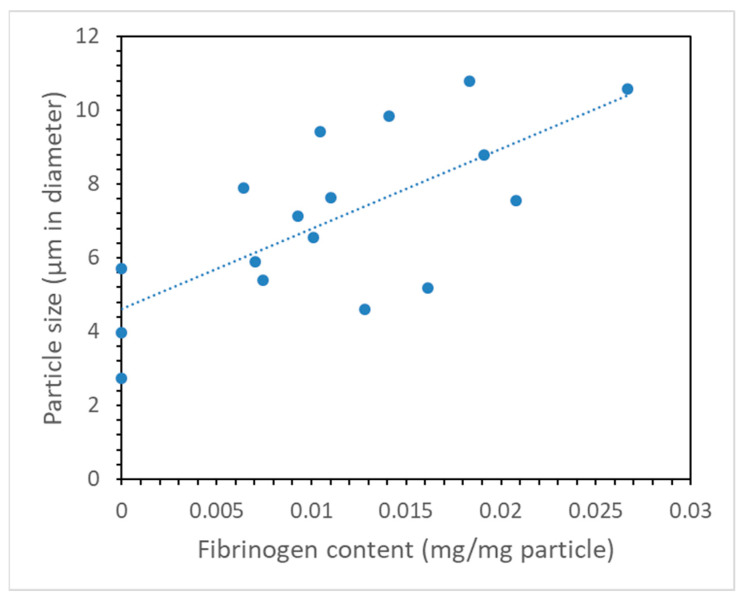
Effects of fibrinogen content on the size of fibrinogen-encapsulated CaCO_3_ particles. A linear relationship with a Pearson correlation coefficient of 0.689 (*p* = 0.002) was observed.

The particles prepared at a higher fibrinogen concentration were larger than those prepared at a lower fibrinogen concentration and their controls with no fibrinogen (Enc AC Fib_high_ Hep versus Enc AC Fib Hep versus Enc AC NoFib Hep). Fibrinogen-containing particles showed a larger size compared to their controls. In particular, the control particles prepared in the absence of fibrinogen (Enc AC NoFib Hep and Enc SC NoFib Hep) were the smallest. Overall, a high correlation between the fibrinogen content and particle size was observed (Figure A1).

Heptane produced larger fibrinogen-containing particles than paraffin, likely due to low viscosity and less stable emulsion when either AC or SC was used as the carbonate source. Increasing the reaction time from 10 to 30 min increased the particle size from 5.196 µm to 6.561 µm. Doubling the amount of surfactants slightly reduced the particle size from 9.416 µm to 8.791 µm (Enc AC_low_ Fib Hep versus Enc AC_low_ Fib Hep Surf_high_), which is consistent with the literature [41]. The addition of PEG increased the particle size from 9.853 µm to 10.565 µm (Enc AC Fib Hep versus Enc AC Fib Hep PEG), while PVA reduced the particle size, perhaps due to its action as an emulsion stabilizer [42]. Switching the inner water phase from AC to CaCl_2_ reduced the particle size from 9.416 µm to 7.561 µm (Enc AC_low_ Fib Hep versus Enc CaCl_2_ Fib Hep AC).

**Figure 2:**
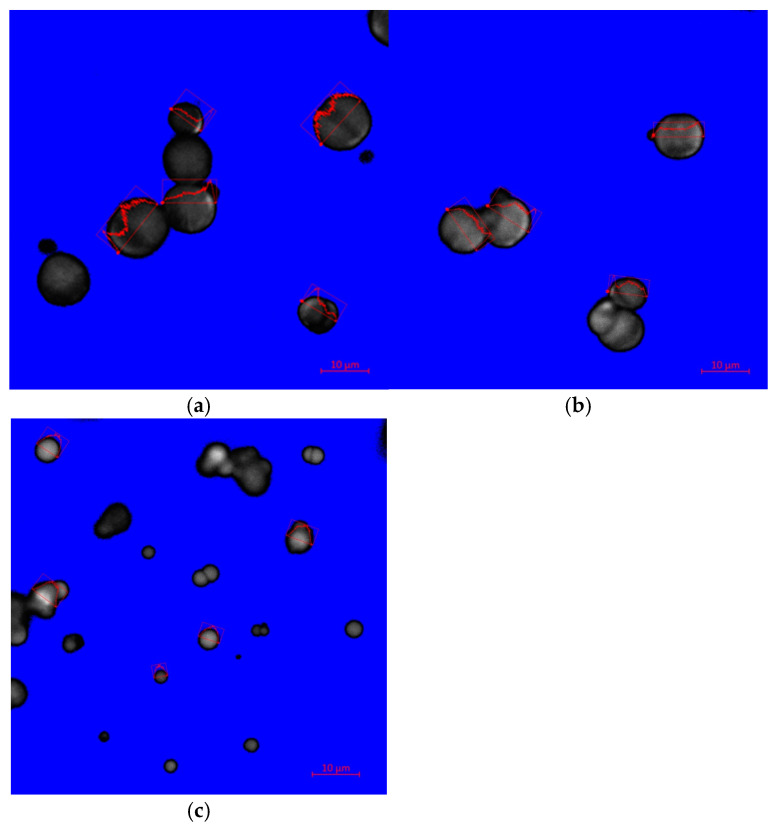
Images of fluorescence microscopy of FITC-labeled fibrinogen-CaCO_3_ particles after subtraction of control particles: (**a**) Enc AC Fib_high_ Hep, (**b**) Enc AC Fib_high_ Par, and (**c**) Enc SBC Fib_high_ Hep. Each particle was prepared under the same conditions as detailed in Table 1 after FITC-labeling of fibrinogen. The scale bar represents 10 µm.

Distinct patterns with the distribution of fibrinogen inside each particle and a dense CaCO_3_ crust were observed from the fluorescent images, showing the brightest cores and dark shells (Figure 2). The AC-produced particle using paraffin as the oil phase showed more centered and uniform fibrinogen distribution within the particle compared to that prepared with heptane (Figure 2b versus Figure 2a). The latter was characterized by dark areas across the particle center (Figure 2a). The SBC-produced particle with heptane as the oil phase showed a centered distribution of fibrinogen (Figure 2c). In particular, the center part of the particle had a stronger fluorescent emission than other places. However, there was a larger variation in fluorescent intensity and particle size than the AC-produced particles (Figure 2c versus Figure 2a,b). It should be noted that the FITC labeling is solely for observing the distribution and location of fibrinogen as it may affect particle production and properties (particle size and fibrinogen content).

### 3.3. Gel Electrophoresis

**Figure 3:**
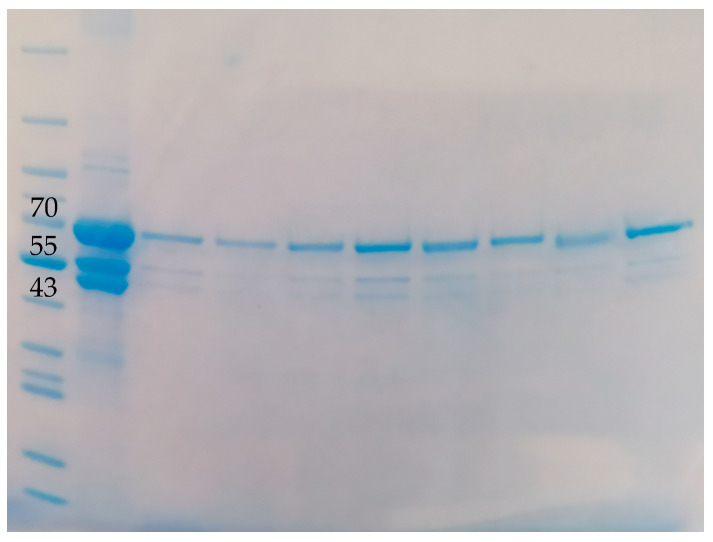
Coomassie-stained SDS-PAGE analysis of fibrinogen and fibrinogen-encapsulated CaCO_3_ particle samples dissolved in TXA^+^ solution. Each sample except standard proteins (Mw STD) was reduced with 5% dithiothreitol and analyzed by the gel electrophoresis. Indicated molecular weights were estimated by Mw STD with known molecular weights from 11 to 250 kDa. From left to right, each lane represents: Mw STD, fibrinogen (5 mg/mL), Enc AC Fib_high_ Par, Enc AC Fib Par, Enc AC Fib_high_ Par 30 min, Enc AC Fib_high_ Hep, Enc AC Fib Hep, Enc AC_low_ Fib Hep, Enc AC_lowest_ Fib Hep, Enc CaCl_2_ Fib Hep AC. See Table 1 for the details of each sample.

Gel electrophoresis was employed to detect fibrinogen. As shown in Figure 3, the initial sample of fibrinogen analyzed by SDS-PAGE under reducing conditions exhibited the typical triplet of bands corresponding to α-, β- and γ-chains of fibrinogen, with molecular weights of approximately 64 kDa, 56 kDa, and 47 kDa, respectively [43].

All fibrinogen-encapsulated CaCO_3_ particles exhibited a relatively strong band of α-chain around 64 kDa, with some particles also showing faint bands corresponding to the β and γ chains at approximately 56 and 47 kDa, respectively. This suggests the presence of fibrinogen within the particles.

As expected, the fibrinogen standards at a higher concentration and particles prepared with a higher fibrinogen concentration (Enc AC Fib_high_ Par versus Enc AC Fib Par and Enc AC Fib_high_ Hep versus Enc AC Fib Hep) showed relatively more intense bands corresponding to fibrinogen α-chain around the 70 kDa mark, along with distinguishable β and γ chain bands. The highest AC concentration (3 M) resulted in more fibrinogen content compared to the lower concentrations at 2 and 1 M (Enc AC Fib Hep versus Enc AC_low_ Fib Hep and Enc AC_lowest_ Fib Hep). Additionally, switching the internal water phase from AC to CaCl_2_ increased band intensity, while extending the reaction time did not enhance fibrinogen encapsulation.

The amount of encapsulated fibrinogen was estimated based on a linear relationship between the fibrinogen standards with known concentrations and the band area of the α-chain, which displayed the strongest density, as shown in Figure 3 and reported in the literature [43,44] (Table 1).

### 3.4. Hemostatic Properties

To further evaluate the presence and functionality of fibrinogen within the particles, ROTEM tests were conducted using human plasma with an abnormally low fibrinogen level. The goal was to observe any changes in the hemodynamic viscoelastic properties of the plasma, measured by CT and MCF, when exposed to the particles.

All fibrinogen-CaCO_3_ particles promoted hemostasis, evidenced by shortened CT and increased MCF compared to control particles prepared by the same method without fibrinogen (Table 2). Notably, the control particles exhibited minimal coagulation, as indicated by non-detectable CT and MCF.

The particles prepared with AC at different concentrations and heptane as the oil phase resulted in the same MCF of 4 mm and a comparable CT between 911 and 1312 s. In comparison, the particles prepared with higher fibrinogen concentration and paraffin as the oil phase (Enc AC Fib_high_ Par and Enc AC Fib_high_ Par 30 min) showed larger hemostatic effects as indicated by shorter CTs (661 and 551 s) and larger MCF (5 mm), respectively.

Furthermore, SC- and SBC-produced particles outperformed AC-produced particles for the hemostatic effects. When either AC or SC was used, paraffin-produced particles showed better hemostatic effects than heptane-produced particles as indicated by larger MCFs (5–6 mm versus 4–5 mm), although the former’s fibrinogen content was not higher.

Doubling the amount of surfactants, the addition of PEG and PVA, and switching the internal water phase did not significantly change the hemostatic effects of the resultant particles.

**Figure A2:**
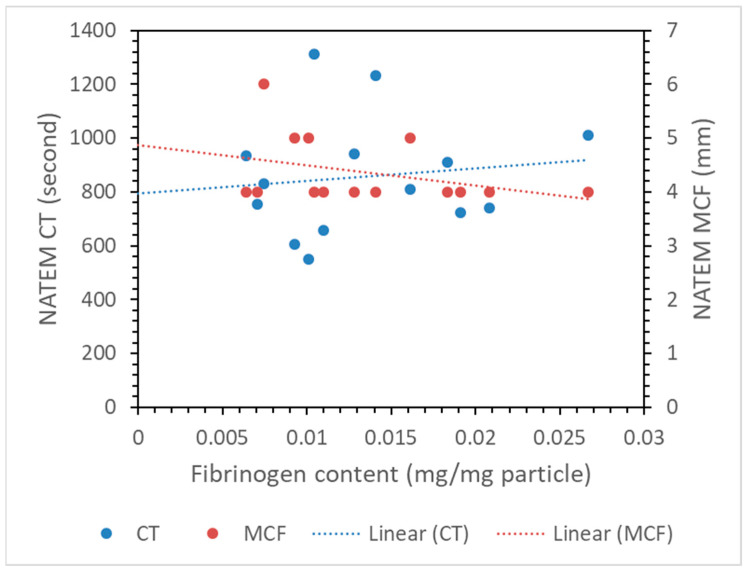
Effects of fibrinogen content on ROTEM coagulation time (CT) and maximum clot firmness (MCF). ROTEM NATEM tests were performed with human plasma containing an abnormally low level of fibrinogen. It should be noted that all control particles prepared in the absence of fibrinogen resulted in non-detectable coagulation and were omitted. The Pearson correlation coefficient between the fibrinogen content and NATEM CT: r = 0.128 (*p* = 0.66), and NATEM MCF: r = −0.354 (*p* = 0.22).

**Figure A3:**
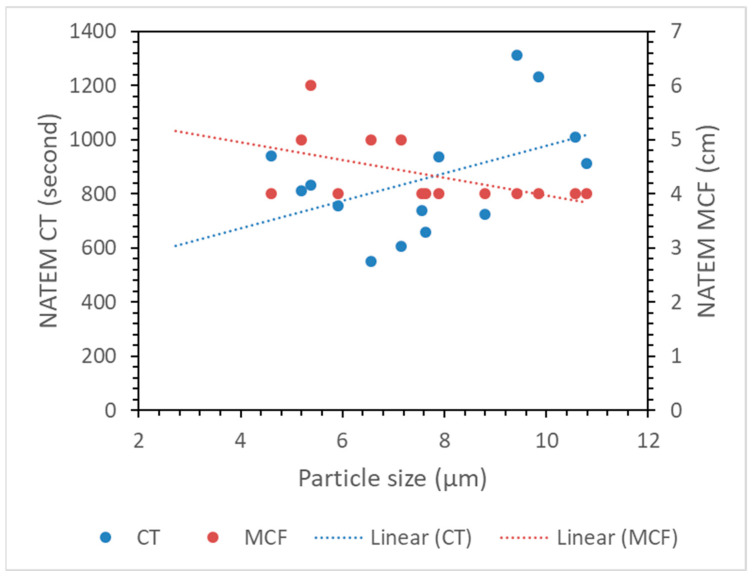
Effects of particle size on ROTEM coagulation time (CT) and maximum clot firmness (MCF). ROTEM NATEM tests were performed with human plasma containing an abnormally low level of fibrinogen. It should be noted that all control particles prepared in the absence of fibrinogen resulted in non-detectable coagulation and thus were omitted. The Pearson correlation coefficient between the particle size and CT: r = 0.466 (*p* = 0.097), and MCF: r = −0.523 (*p* = 0.055).

Overall, the hemostatic effects were not associated with the fibrinogen content as determined by gel electrophoresis (Figure A2), but close to being significantly related to the particle size, suggesting the larger fibrinogen-encapsulated particle likely led to longer CT and smaller MCF (Figure A3).

**Figure 4:**
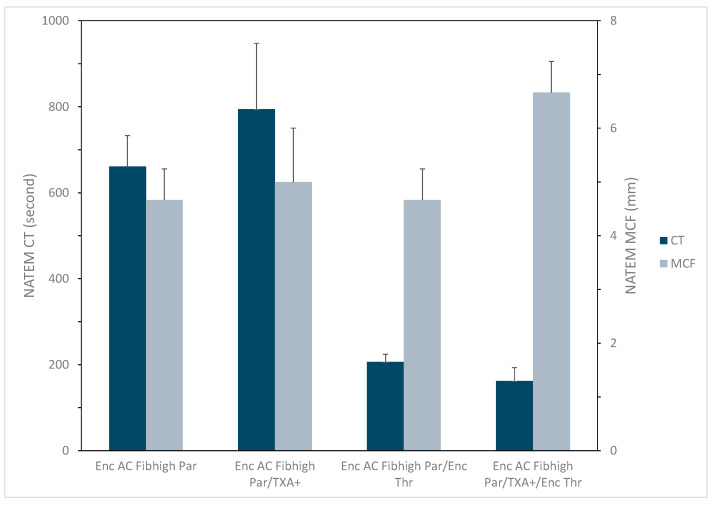
Effects of fibrinogen-encapsulated CaCO_3_ particle (Enc AC Fib_high_ Par) alone and in combination with TXA^+^ and thrombin-encapsulated CaCO_3_ particle (Enc Thr) on ROTEM coagulation time (CT) and maximum clot firmness (MCF). ROTEM NATEM tests were performed with plasma containing an abnormally low level of fibrinogen in the presence of 6 mg Enc AC Fib_high_ Par alone and together with 2 mg TXA^+^ and 2 mg Enc Thr (see Section 2.3.3 for details). Data represent mean ± SD (n = 3).

As shown in Figure 4, combining fibrinogen-CaCO_3_ particle with TXA^+^ did not lead to significant changes in CT and MCF. The fibrinogen-CaCO_3_ particle mainly increased MCF, while the thrombin-CaCO_3_ particle remarkably decreased CT when they were mixed with plasma. Interestingly, the largest hemostatic effect was achieved when the fibrinogen- and thrombin-encapsulated CaCO_3_ particles were combined with TXA^+^ as indicated by both the shortest CT (162.2 s) and largest MCF (6.7 mm) (Enc AC Fib_high_ Par/TXA^+^/Enc Thr in Figure 4).

### 3.5. Hemolytic Activities

The in vitro hemolysis test is a widely used method to assess the hemocompatibility of biomaterials [45]. As summarized in Table 1, different particle samples exhibited varying levels of hemolysis (hemoglobin release), all of which were well below the permissible threshold for biomaterials (<5%) [46], demonstrating favorable blood compatibility. In fact, many of the particles showed negative hemolysis values, suggesting they may prevent hemolysis. The excellent compatibility with RBCs indicates that these particles could be promising candidates for in vivo applications.

### 3.6. Self-Propelling Properties

The self-propelling properties of the mixed CaCO_3_ particles and TXA^+^ were measured at a mass ratio of 1.2, as previously determined [37]. The self-propulsion test assesses the particles’ capability to deliver fibrinogen against blood flow to a bleeding site. All particles demonstrated self-propulsion when mixed with TXA^+^ and made contact with water, exhibiting varying lag times and speeds (Table 2).

**Figure A4:**
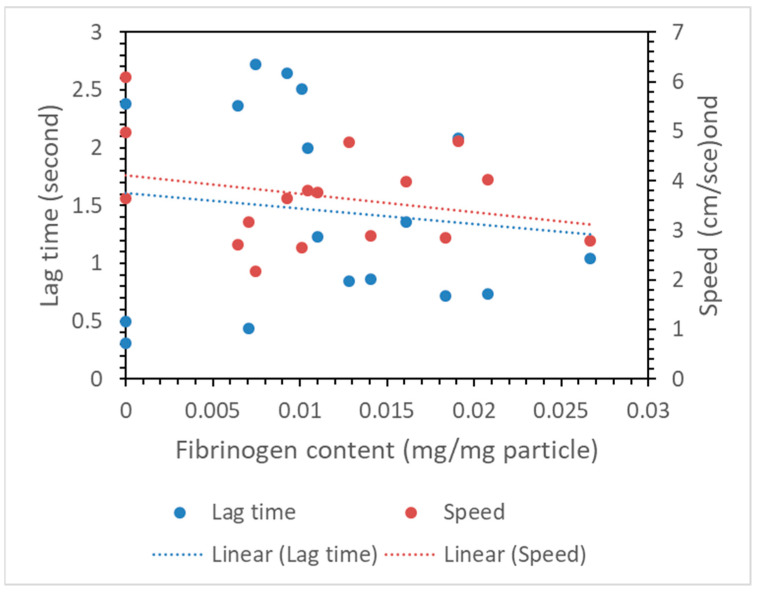
Effects of fibrinogen content on the self-propelling lag time and speed of fibrinogen-CaCO_3_ particles. No linear association of the fibrinogen content with the lag time and speed was observed: r = −0.119 (*p* = 0.65) and r = −0.276 (*p* = 0.28), respectively.

**Figure A5:**
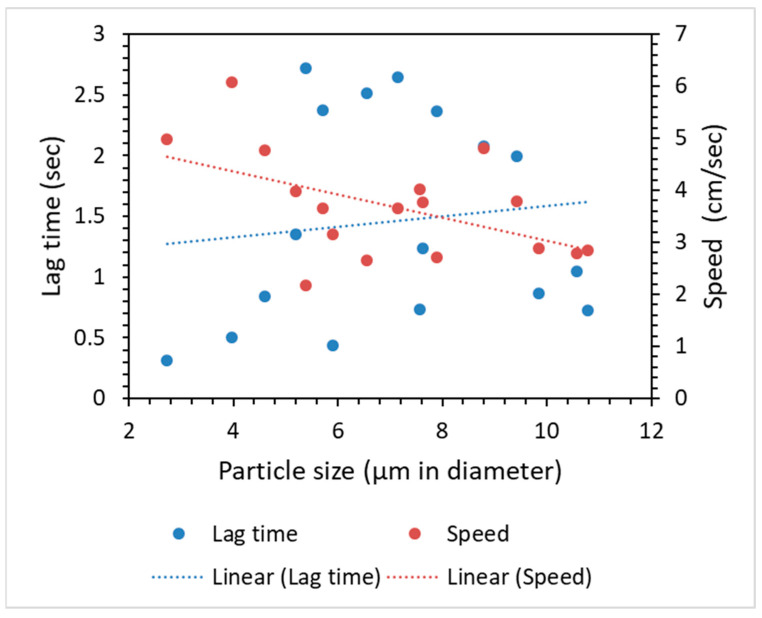
Effects of particle size on the self-propelling lag time and speed of fibrinogen-CaCO_3_ particles. No linear relationship between the particle size and self-propelling lag time r = 0.117 (*p* = 0.65), and a linear relationship between the particle size and self-propelling speed with a Pearson correlation coefficient of −0.506 (*p* = 0.038) was observed.

It appeared that the carbonate source and fibrinogen content did not affect the self-propelling properties as measured by the lag time and propelling speed (Figure A4), although the control particles (Enc AC NoFib Hep and Enc SC NoFib Hep) showed the shortest lag time and fastest speed. The particle prepared with the lowest concentration of AC (Enc AC_lowest_ Fib Hep), 1 M, exhibited a shorter lag time and faster speed than those obtained at higher concentrations, such as 2 M and 3 M. The heptane-produced particles exhibited a lag time shorter than 1.5 s and a better self-propelling ability than those prepared with paraffin as the oil phase (with a lag time longer than 2 s). Increasing the surfactant amount did not shorten the lag time, but increased the propulsion speed (Enc AC_low_ Fib Hep Surf_high_ versus Enc AC_low_ Fib Hep). The addition of PVA reduced the lag time from 0.844 to 0.439 s and increased the speed from 2.892 to 3.16 cm/second, while addition of PEG did not significantly affect the self-propelling performance. Switching the internal water phase improved the self-propulsion as well, with shorter lag time and faster speed (Enc CaCl_2_ Fib Hep AC versus Enc AC_lowest_ Fib Hep). Overall, only self-propelling speed was highly associated with the particle size, suggesting a faster speed for a smaller particle (Figure A5).

## 4. Discussion

CaCO_3_ particles find diverse applications, such as in medicine and drug delivery [47,48,49,50], and delivery of procoagulants against blood flow in combination with TXA^+^ [28,51]. With an anamorphous phase and three distinct polymorphs: vaterite, aragonite and calcite, in order of increasing thermodynamic stability, and intricate crystallization behavior, achieving precise control over the physical properties of CaCO_3_ particles for specific uses is important [52,53]. The key finding of this study is the feasibility of the encapsulation of large coagulation factor fibrinogen into CaCO_3_ particles and the retention of hemostatic functions. Additionally, the fibrinogen-encapsulated CaCO_3_ particles showed self-propulsion when mixed with TXA^+^. The preparation conditions (e.g., carbonate source) affected yield, particle size, morphology, amount of encapsulated fibrinogen, hemostatic and self-propelling properties. It is hypothesized that the reaction between calcium and carbonate ions might occur around the interface between the fibrinogen aqueous solution droplet and the oil membrane, resulting in the precipitation of CaCO_3_ on the exterior and the encapsulation of fibrinogen within the particle. Further investigation of this phenomenon is warranted.

While previous studies have successfully demonstrated the encapsulation of various biomacromolecules in CaCO_3_ using a W/O/W emulsion [54,55], the largest molecule encapsulated has been bovine serum albumin, with a molecular weight of 66 kDa—significantly smaller than that of fibrinogen, which is 340 kDa. On the other hand, it was reported that the encapsulation efficiency of proteins decreased with their molecular weights; small molecules, such as lysozyme (14,388 Da) diffused to the outer water phase too quickly to be included in the forming CaCO_3_ particle along the W/O/W emulsion interface [54].

On the other hand, alternative encapsulation techniques, such as lipid particles systems have been explored in previous hemostatic research. For example, thrombin-loaded injury-site-targeted lipid nanoparticles have been developed to augment hemostasis in coagulopathic bleeding [56]. However, the W/O/W approach uniquely accommodates larger proteins like fibrinogen, offering advantages in stability and efficacy under physiological conditions. In this study, we further adopted our previous process for synthesizing fibrinogen- and thrombin-encapsulated CaCO_3_ particles [33] and investigated the effects of preparation conditions on the particle yield, fibrinogen encapsulation, particle size, morphology, hemostatic and self-propelling properties. It is anticipated that multiple factors contribute to the observed effects in each sample. One potential factor could be reaction kinetics between carbonate and CaCl_2_ and the stability of fibrinogen during encapsulation due to the presence of the oil phase. Several experimental parameters could affect the W/O/W double emulsion system and thus particle size, fibrinogen content, and function, such as type of oil, and amount of surfactant. The latter could also determine the crystalline structure of CaCO_3_ [57].

Large porosity and specific surface area are usually favorable for hemostatic microparticles containing biomacromolecules [23] and can be controlled by the types of carbonate and calcium ions of material sources, and inner and outer water phases in the preparation of CaCO_3_ particles by the interfacial reaction method using W/O/W emulsion [54].

The type of carbonate salts plays an important role in the production of CaCO_3_, protein encapsulation, secondary structure, and bioactivity, in agreement with the literature [54]. The highest yield obtained with SC as the carbonate source may be ascribed to its fastest reaction with CaCl_2_ compared to AC and SBC. The larger SC-produced particle compared to the AC-produced particle is consistent with that produced with K_2_CO_3_ using the same method [54]. On the other hand, as most biomacromolecules seem to be denatured in the high alkaline solution, AC was employed instead of K_2_CO_3_ in the cases of biomacromolecule encapsulation [54]. Similarly, AC and SBC were used as carbonate precursors in our study to generate a lower final pH (8.8 and 7.7) for reconstituted fibrinogen solution than that using SC (10.5) in order to preserve the native structure of fibrinogen [58]. It is well known that solution pH could result in functional loss of fibrinogen [59,60]. By a slow addition of fibrinogen reconstituted in a HEPES solution to an SC solution, a maximum concentration of 1 M SC could be added to the fibrinogen-HEPES solution without inducing fibrinogen precipitation. For SBC, its limited solubility in water (20 °C) at 96 mg/mL posed a major constraint on the concentration that could be used. The highest concentration investigated was also 1 M, corresponding to about 84 mg/mL, approximately 87.5% of its solubility capacity. Reconstitution of fibrinogen in an AC solution could be successfully performed at concentrations varied from 1 M to 3 M. Alternatively, fibrinogen was reconstituted in CaCl_2_ as the inner water phase was close to a neutral pH (Enc CaCl_2_ Fib Hep AC), avoiding the possible reduction in its bioactivity under alkaline conditions in the carbonate solutions, and leading to a higher yield, fibrinogen content, better hemostatic and self-propelling properties compared to that obtained with AC as the inner water phase at the same concentration (Enc AC_low_ Fib Hep).

Fibrinogen encapsulation and distribution inside CaCO_3_ particle was confirmed by fluorescence microscopy of FITC-labeled samples and the particle size observed in our study was in the same range as that of other protein-encapsulated CaCO_3_ particles [54], but larger than the CaCO_3_ hollow capsules ranging from 0.5 to 2 µm prepared in similar W/O/W double emulsions (no encapsulated molecules) [41]. This is also in agreement with the increased particle size with increasing fibrinogen concentration from 0 to 20 mg/mL and high positive correlation between the fibrinogen content and particle size as depicted in Figure A1.

The amorphous, vaterite, and calcite phase structures of CaCO_3_ depend on preparation methods and conditions. The interfacial reaction method using W/O/W emulsion is considered to produce hollow CaCO_3_ spherical particles and microcapsules encapsulating proteins with main crystalline phase of vaterite that is a metastable phase of crystalline CaCO_3_ [41,54]. The transition from the vaterite phase to the calcite phase occurred when the microcapsules were aged in some aqueous solutions and further encapsulation of proteins could be obtained through the phase transition from vaterite to calcite when left in protein aqueous solutions for a few days [61]. Therefore, the reaction rate of CaCO_3_ with TXA^+^ which affects self-propulsion and release of procoagulants, could be adjusted by controlling the crystal phase of CaCO_3_ [62]. On the other hand, mutual effects may exist in the crystallization of CaCO_3_ particles and the secondary structure of proteins [63]. The effect of various additives (PEG, PVA) on the formation, nucleation process and growth of CaCO_3_ particles is very complicated and rather unpredictable [34,62,63].

The effect of the oil phase is most likely attributed to its capacity to modify the morphology of CaCO_3_ particles and functionality of fibrinogen. When exposed to heptane or paraffin, fibrinogen could undergo unfolding or denaturation, primarily driven by interactions with its hydrophobic regions. These alterations could also affect the uniformity of the encapsulated particles, as the W/O/W emulsion plays a pivotal role in stabilizing and ensuring the consistent size of the particles produced.

We undertook a qualitative and relatively quantitative analysis of encapsulated proteins using gel electrophoresis which quantified plasma proteins in particular fibrinogen adsorbed on hemostatic dressings based on its band density [44]. However, gel electrophoresis does not differentiate between functional and non-functional fibrinogen. In contrast, the coagulation functional assay [64], which assesses the biological function of the molecule, serves as a much more sensitive indicator of retention of its native state than structural probes [65].

CT and MCF are two commonly used ROTEM parameters, primarily influenced by plasma clotting factors and reflecting the dynamic properties of fibrin as well as platelet number and function, respectively [66]. Human plasma with an abnormally low fibrinogen level was used to mimic conditions, where fibrinogen supplementation would demonstrate hemostatic effects. Consistently, all fibrinogen-encapsulated particles led to improved plasma coagulation to various extents.

Given the limited dissolution of CaCO_3_ particles in plasma at neural pH, the encapsulated fibrinogen might not be completely released during ROTEM measurement to impose full hemostatic effects. Additionally, the fibrinogen content as estimated by gel electrophoresis included both active and inactive fibrinogen. Together, it may result in no correlation between the fibrinogen content and the hemostatic effects as measured by NATEM CT and MCF (Figure A2).

PEG and PVA are commonly used in wound hemostasis therapy [67]. It has been shown that low concentrations of 5–10 g/L of PEG with a molecular weight of 6000 markedly enhance the clotting of fibrinogen solutions with thrombin resulting from increased fibrin polymerization and crosslinking [68]. PVA could further stabilize the emulsion [42]. However, no significant increase in the hemostatic effects was observed with the addition of PEG and PVA in the particle preparation. On the other hand, larger hemostatic effects were achieved when the particles were combined with TXA^+^ and thrombin-encapsulated CaCO_3_.This likely results from enhanced particle dissolution and the subsequent release of coagulation factors, leading to synergistic effects between fibrinogen, thrombin and TXA on hemostasis. The combined action of fibrinogen- and thrombin-loaded CaCO_3_ particles suggests that this approach could reduce coagulation time, strengthen clot formation, and ultimately provide a more efficient method for hemorrhage control. Furthermore, as it is possible for encapsulation of different coagulation factors inside CaCO_3_ particles and pre-mixed them together in a dry form without reaction until coming into contact with blood to achieve synergistic hemostatic effects.

Particle size may have a significant impact on the hemostatic effect [23]. It was reported that particle size over a range of <100–500 nm had a significant impact on particle-platelet interactions [69]. However, given its narrow range in micrometers (4–10 µm), the particle size only had a trend toward remarkable impact on hemostatic effects on plasma in our study (Figure A3).

Furthermore, as a simple and reliable measure for estimating blood compatibility of biomaterials, the hemolysis analysis demonstrated that the hemostatic particles were compatible with human erythrocytes, supporting their further development for hemorrhage control.

The self-propulsion is a measure of the capability that the particle has to deliver procoagulants against blood flow to a bleeding site. The property was retained in all particles and might be affected by the preparation conditions, with smaller particles showing faster speed. Because of the multiple mechanisms of propulsion and the complex nature of blood flow (turbulent or pulsating flow and heterogeneous solutions) in wounds, particles were not expected to maintain their velocity in a single direction as observed in our in vitro test [35].

The self-propelling properties of CaCO_3_ particles were not compromised when loaded with fibrinogen, as indicated by the lack of association between fibrinogen content and both self-propelling lag time and speed. In contrast, fibrinogen-CaCO_3_ particles prepared by absorption and precipitation methods exhibited longer lag times (4.011–15.218 s) and slower speeds (1.286–3.067 cm/second), particularly for those lyophilized with the fibrinogen solution, as previously reported [37]. This suggests the encapsulation method resulted in superior self-propelling properties, characterized by shorter lag times and faster movement.

These findings provide a basis for further development of fibrinogen-encapsulated CaCO_3_ with desired properties for hemorrhage control. A more quantitative analysis of the coagulation factor encapsulation with CaCO_3_ could be performed using coagulation functional assay [64] and enzyme-linked immunosorbent assay [70]. Although CaCO_3_ has long been used as a delivery system for bioactive compounds to improve their safety and efficacy given its wide availability, biocompatibility, and degradability [48], cytotoxicity and microbiological tests should be conducted in future studies to further characterize this novel hemostatic particle. On the other hand, our previous studies on self-propelling particles composed of thrombin-containing CaCO_3_ and TXA^+^ have shown no adverse local and systemic effects in small and large animal bleeding models, as confirmed by histological analyses [35,71,72]. Combined with other studies on the cytotoxicity and hemolysis of bioactive compound-encapsulated CaCO_3_ particles [73,74], it is expected that the fibrinogen-encapsulated CaCO_3_ particle would be biocompatible. However, additional work is required to rigorously evaluate the safety and toxicity of these newly formulated particles for specific applications. Moreover, characterization with other analytical techniques (e.g., Fourier-transform infrared spectroscopy, electronic microscopy and X-ray diffraction) would provide chemical and physical structures of the resultant particles to further optimize their properties. Continuing this research is warranted due to the simple preparation process, the wide availability and low cost of raw materials, and the promising properties of the resulting hemostatic particles. The primary production cost would stem from the use of medical-grade fibrinogen, which is priced at approximately $400 per gram [75].

## 5. Conclusions

It is feasible to produce fibrinogen-encapsulated CaCO_3_ particles in micrometer size with hemostatic and self-propelling properties by the W/O/W emulsion method. Fibrinogen encapsulation was affected by carbonate source, concentration, fibrinogen concentration, type of oil and internal water phase. The self-propelling particles composed of fibrinogen- and thrombin-CaCO_3_ particles and TXA^+^ showed synergistic hemostatic effects. Further investigation on optimal formulation and hemostatic effects of the self-propelling particles composed of fibrinogen-CaCO_3_ particles in an animal bleeding model is warranted.

## Figures and Tables

**Table 1:** Preparation of fibrinogen-encapsulated CaCO_3_ particles by the W/O/W emulsion method under various conditions.

Sample ID ^1^	Carbonate Source and Concentration (mol/L)	Fibrinogen Concentration in Carbonate Solution (g/L)	Oil phase and Volume (mL)	Yield (%) ^2^	Encapsulated Fibrinogen mg/mg Particle
Enc AC_lowest_ Fib Hep	AC 1	15	Heptane 14	36	0.0128
Enc AC_low_ Fib Hep	AC 2	15	Heptane 14	40	0.0105
Enc AC Fib Hep	AC 3	15	Heptane 14	50	0.0141
Enc SC Fib Hep	SC 1	15	Heptane 14	79	0.0110
Enc AC Fib_high_ Hep	AC 3	20	Heptane 14	39	0.0183
Enc SBC Fib_high_ Hep	SBC 1	20	Heptane 14	21	0.0161
Enc AC Fib Par	AC 3	15	Paraffin 14	43	0.0064
Enc AC Fib_high_ Par	AC 3	20	Paraffin 14	41	0.0093
Enc AC Fib_high_ Par 30 min	AC 3	20	Paraffin 14	45	0.0101
Enc AC NoFib Hep ^3^	AC 3	0	Heptane 14	51	0
Enc SC NoFib Hep ^3^	SC 1	0	Heptane 14	81	0
Enc AC NoFib Par 30 min ^3^	AC 3	0	Paraffin 14	46	0
Enc SC Fib Par	SC 1	15	Paraffin 14	77	0.0075
Enc AC_low_ Fib Hep Surf_high_ ^4^	AC 2	15	Heptane 14	43	0.0191
Enc AC Fib Hep PEG	AC 3 + 15 mg/mL PEG	15	Heptane 14	39	0.0267
Enc-AC Fib Hep PVA	AC 3 + 80 mg/mL PVA	15	Heptane 14	42	0.0071
Enc CaCl_2_ Fib Hep AC ^5^	2 M CaCl_2_	15	Heptane 14	70	0.0208

^1^ Each sample was named as Enc for encapsulation, internal water phase (AC for ammonium carbonate, SC for sodium carbonate, SBC for sodium bicarbonate), Fib for fibrinogen, oil phase (Hep for heptane and Par for paraffin), Surf for surfactant, and additional polymers (PEG for polyethylene glycol and PVA for polyvinyl alcohol), and subscripts for high and low concentrations; ^2^ Calculated as the actual amount of product divided by the sum of theoretical amount of CaCO_3_ that should be produced and initial amount of added fibrinogen in the preparation; ^3^ Control particles, i.e., CaCO_3_ particles in the absence of fibrinogen; ^4^ Doubled amount of Span80/Tween 80; ^5^ CaCl_2_ as internal water phase at 2 M and AC as outer water phase at 0.3 M.

**Table 2:** The size, hemostatic, hemolytic and self-propelling properties of fibrinogen-encapsulated CaCO_3_ particles prepared by the emulsion method under different conditions.

Sample ID ^1^	Particle Size in Diameter (µm, n = 10)	NATEM			Self-Propelling	
		CT (Second) ^2^	MCF (mm) ^3^	Hemolysis (%)	Lag Time (Second) (n = 10)	Speed (cm/Second) (n = 10)
Enc AC_lowest_ Fib Hep	4.607 ± 0.519	941	4	−1.198	0.844 ± 0.084	4.779 ± 0.613
Enc AC_low_ Fib Hep	9.416 ± 1.089	1312	4	0.005	1.999 ± 0.296	3.798 ± 1.357
Enc AC Fib Hep	9.853 ± 0.532	1232	4	0.009	0.866 ± 0.145	2.892 ± 0.737
Enc SC Fib Hep	7.629 ± 1.519	659	4	−0.740	1.233 ± 0.219	3.761 ± 1.203
Enc AC Fib_high_ Hep	10.792 ± 0.724	911	4	−1.089	0.722 ± 0.184	2.852 ± 0.791
Enc SBC Fib_high_ Hep	5.187 ± 0.975	811	5	−9.087	1.355 ± 0.259	3.988 ± 0.648
Enc AC Fib Par	7.896 ± 0.729	936	4	−9.380	2.367 ± 1.114	2.710 ± 0.607
Enc AC Fib_high_ Par	7.141 ± 1.035	661.3 ± 71.7	4.7 ± 0.6	−9.676	2.647 ± 0.484	3.653 ± 0.651
Enc AC Fib_high_ Par 30 min	6.561 ± 0.852	551	5	−11.358	2.511 ± 0.184	2.659 ± 0.767
Enc AC NoFib Hep	2.731 ± 0.524	Not detectable		−2.739	0.311 ± 0.287	4.977 ± 2.354
Enc SC NoFib Hep	3.975 ± 1.573	Not detectable		−5.870	0.500 ± 0.202	6.082 ± 2.186
Enc AC NoFib Par 30 min	5.703 ± 0.894	Not detectable		−4.304	2.377 ± 0.157	3.653 ± 1.031
Enc SC Fib Par	5.384 ± 1.655	831	6	−0.784	2.721 ± 0.407	2.174 ± 0.522
Enc AC_low_ Fib Hep Surf_high_	8.791 ± 1.142	723	4	−0.260	2.078 ± 1.058	4.801 ± 2.627
Enc AC Fib Hep PEG	10.565 ± 2.466	1010	4	0.014	1.044 ± 0.434	2.787 ± 0.507
Enc AC Fib Hep PVA	5.906 ± 1.640	755	4	0.010	0.439 ± 0.117	3.160 ± 1.859
Enc CaCl_2_ Fib Hep AC	7.561 ± 1.321	739	4	−2.034	0.733 ± 0.524	4.016 ± 1.937

^1^ Each sample was named as Enc for encapsulation, internal water phase (AC for ammonium carbonate, SC for sodium carbonate, SBC for sodium bicarbonate), Fib for fibrinogen, oil phase (Hep for heptane and Par for paraffin), Surf for surfactant, and additional polymers (PEG for polyethylene glycol and PVA for polyvinyl alcohol), and subscripts for high and low concentrations. ^2^ CT for coagulation time. ^3^ MCF for maximum clot firmness. See Table 1 for particle preparation in detail.

## Data Availability

The original contributions presented in the study are included in the article, further inquiries can be directed to the corresponding author.
